# Anti-asthmatic prescriptions in children with and without congenital anomalies: a European data linkage study.

**DOI:** 10.23889/ijpds.v7i3.1889

**Published:** 2022-08-25

**Authors:** Natalie Divin, Ester Garne, Joan K Morris, Maria Loane

**Affiliations:** 1 Ulster University; 2 Pediatric Department, Hospital Lillebaelt Kolding; 3 St George’s University of London

## Objectives

Asthma is the most common chronic disease in childhood, yet little is known about rates of asthma and wheezing in children with congenital anomalies. This study explored the prevalence and risk of receiving anti-asthmatic prescriptions in children with congenital anomalies compared to children without anomalies in six European regions/countries.

## Approach

This was a EUROlinkCAT population-based linkage cohort study involving children from 0-9 years of age born between 2000-2014. Congenital anomaly data from six EUROCAT registries were linked to births data in national/vital statistics and to electronic prescription databases. Prescription/pharmacy dispensing records across regions were standardised to a Common Data Model. Anatomical Therapeutic Chemical classification codes beginning with R03 were used to identify anti-asthmatic prescriptions. Random-effects meta-analyses were performed to identify both the relative risk (RR) of receiving >1 anti-asthmatic prescription in a year relative to the reference group, and the heterogeneity of prevalence rates across registries and age group.

## Results

A total of 5.1% of children with congenital anomalies and 4.9% of reference children were dropped from the study as they were not linked. Children with congenital anomalies (n=60,662) had a higher prevalence of >1 anti-asthmatic prescription and a significantly higher risk of being prescribed anti-asthmatics (RR=1.41, 95% CI 1.35-1.48) compared to reference children (n=1,722,912). The increased risk was consistent across all age groups. Children with congenital anomalies were more likely to be prescribed beta-2 agonists (RR=1.71, 95% CI 1.60-1.83) and inhaled corticosteroids (RR=1.74, 95% CI 1.61-1.87). Children with oesophageal atresia, diaphragmatic hernia, genetic syndromes and chromosomal anomalies had over twice the risk of being prescribed anti-asthmatics compared to reference children. Regional differences in prevalence and risk of anti-asthmatic prescriptions were identified.

## Conclusion and Relevance

Children aged <10 years with congenital anomalies consistently had higher prevalence and risk of receiving >1 anti-asthmatic prescription across age group and across European regions. This study demonstrates that information on the prevalence of anti-asthmatic prescriptions issued/dispensed can be obtained through data linkage to monitor changes in prevalence over time.

